# Draft Genome Sequence of *Janthinobacterium* sp. Strain PC23-8, Isolated from a Freshwater Stream Impacted by Acid Mine Drainage

**DOI:** 10.1128/MRA.00850-19

**Published:** 2019-10-10

**Authors:** Caitlin E. Clark, Matthew G. Banyas, Stephen E. Noell, David A. Essig

**Affiliations:** a Department of Biology, Geneva College, Beaver Falls, Pennsylvania, USA; University of Maryland School of Medicine

## Abstract

We present the draft genome sequence of *Janthinobacterium* sp. strain PC23-8, a bacterium isolated from freshwater stream sediment downstream from acid mine drainage. The 6.4-Mb genome sequence of this strain contains several secondary metabolite gene clusters, including one similar to the cyclic peptide jagaricin, synthesized by Janthinobacterium agaricidamnosum.

## ANNOUNCEMENT

For genome mining studies aimed at drug discovery, extreme environments may yield less typical bacterial species as sources of novel antimicrobials (1–3). In our study, sediment was collected from Davidson Creek (Beaver County, PA, USA) at a site impacted by acid mine drainage. Diluted sediment samples were spread on Trypticase soy agar (TSA) plates to allow formation of single colonies at 25°C. Isolates were colony purified and subjected to 16S rRNA sequencing (Genewiz, South Plainfield, NJ). EzBioCloud was used to find the closest related taxonomic groups (4). Isolate PC23-8 was a nonpigmented, Gram-negative, motile bacillus that showed homology (∼99%) to several well-described *Janthinobacterium* species, including the red-brown-pigmented J. svalbardenesis, the purple-pigmented J. lividum strains, and the nonpigmented *J. agaricidamnosum*.

The genome sequence was generated at ACGT, Inc. (Wheeling, IL). Genomic DNA was isolated from colonies grown on TSA plates using the MasterPure complete DNA/RNA purification kit (Epicentre catalog number MC85200) and purified using the DNA Clean & Concentrator kit (Zymo Research). For paired-end (PE) sequencing, genomic DNA was fragmented using ultrasonication (∼500 bp) and used to construct a library with a NEXTflex rapid DNA-Seq library preparation kit (Bioo Scientific). Genomic DNA was also used to prepare a Nextera mate pair (MP; 4- to 10-kb) library. Final fragmentation of the library was performed using ultrasonication to a size of ∼500 bp. Both libraries were size selected for 600 to 800 bp using the BluePippin system (Sage Science, Beverly, MA) and verified with a 2100 bioanalyzer (Agilent, Santa Clara, CA). The PE and MP libraries were sequenced using HiSeq and NextSeq, respectively.

For bioinformatic analyses, default settings were used unless otherwise specified. Illumina reads were demultiplexed (bcl2fastq v2.17, Illumina, San Diego, CA) and trimmed (Trim Galore v4.1; Babraham Bioinformatics, Cambridge, UK; q = 30, PE lower limit = 50, MP lower limit = 35). Trimmed reads, assembled using SPAdes v3.7.1 (*t* = 56, mismatch = careful) (5), totaled 3,489,090,776 bp for the PE library and 1,390,229,081 bp for the MP library. Sequence coverage was estimated at 766-fold. Contigs shorter than 1,000 bp were discarded. The assembled genome consisted of 12 contigs. The overall size of the draft sequence was 6,386,497 bp with 11 physical gaps. Using RAST v2.0 (6), the *N*_50_ value of the genome assembly was found to be 326,467 bp, and the genome contained 5,600 protein-coding and 93 RNA genes. antiSMASH v5.0 (stringent) (7) predicted one gene cluster to generate a cyclic peptide related to the antifungal jagaricin (8). Other predicted clusters coded for novel peptides, including several bacteriocins and a beta-lactone. Using the Genome-to-Genome Distance Calculator (GGDC; formula 2) (9), PC23-8 was found to be a species distinct from *J. svalbardenesis* (27.5% sequence identity), J. lividum (26.4% sequence identity), and *J. agaricidamnosum* (22.9% sequence identity), since values were less than the same-species threshold (70%).

### Data availability.

This whole-genome shotgun project has been deposited in GenBank under the accession number NZ_NJGY00000000. The version described in this paper is the first version. The SRA accession numbers for the PE and MP libraries used to assemble the genome are SRR10043460 and SRR10043459, respectively.
